# Correction: Single-molecule FRET and conformational analysis of beta-arrestin-1 through genetic code expansion and a Se-click reaction

**DOI:** 10.1039/d1sc90143e

**Published:** 2021-07-02

**Authors:** Ming-Jie Han, Qing-tao He, Mengyi Yang, Chao Chen, Yirong Yao, Xiaohong Liu, Yuchuan Wang, Zhong-liang Zhu, Kong-kai Zhu, Changxiu Qu, Fan Yang, Cheng Hu, Xuzhen Guo, Dawei Zhang, Chunlai Chen, Jin-peng Sun, Jiangyun Wang

**Affiliations:** Tianjin Institute of Industrial Biotechnology, Chinese Academy of Sciences, Tianjin Airport Economic Area Tianjin 300308 China; Key Laboratory Experimental Teratology of the Ministry of Education, Department of Biochemistry and Molecular Biology, School of Basic Medical Sciences, Cheeloo College of Medicine, Shandong University 44 Wenhua Xi Road Jinan 250012 Shandong China sunjinpeng@sdu.edu.cn; School of Life Sciences, Tsinghua-Peking Joint Center for Life Sciences, Beijing Advanced Innovation Center for Structural Biology, Tsinghua University Haidian District Beijing 100084 China chunlai@tsinghua.edu.cn; Department of Physiology and Pathophysiology, School of Basic Medical Sciences, Peking University, Key Laboratory of Molecular Cardiovascular Science, Ministry of Education Haidian District Beijing 100191 China; Institute of Biophysics, Chinese Academy of Sciences Chaoyang District Beijing 100101 China; University of the Chinese Academy of Sciences (UCAS) Shijingshan District Beijing 100049 China; Shenzhen Institute of Transfusion Medicine, Shenzhen Blood Center Futian District Shenzhen 518052 China; School of Life Sciences, University of Science and Technology of China Baohe District Anhui 230026 China; School of Biological Science and Technology, University of Jinan Jinan Shandong 250022 China

## Abstract

Correction for ‘Single-molecule FRET and conformational analysis of beta-arrestin-1 through genetic code expansion and a Se-click reaction’ by Ming-Jie Han *et al.*, *Chem. Sci.*, 2021, DOI: 10.1039/D1SC02653D.

In the ‘Monitoring conformational changes of βarr1 activated by V2Rpp through smFRET’ section of the main article, references 16, 48 and 49 were incorrectly introduced as recent crystallographic and NMR studies on arrestin structure and function. These studies refer instead to cryoEM and serial crystallography studies of arrestin.

The Royal Society of Chemistry apologises for these errors and any consequent inconvenience to authors and readers.

